# 1107. Three Year Longitudinal Assessment of *Pseudomonas aeruginosa* (Pa) Antibiotic Susceptibility in Children with Cystic Fibrosis (CF) in the Era of Modulator Therapy

**DOI:** 10.1093/ofid/ofad500.080

**Published:** 2023-11-27

**Authors:** John Bradley, Dayna Stout, Kathryn Akong, Yaron Fireizen

**Affiliations:** UCSD/RCHSD, San Diego, California; Rady Children's Hospital San Diego, San Diego, CA; Rady children's hospital/University of California San Diego, San Diego, California; Rady children's hospital/University of California San Diego, San Diego, California

## Abstract

**Background:**

CF is a genetic disease characterized by chronic lung infection, often with *Pseudomonas aeruginosa* (Pa). It is widely accepted that increasing antibiotic resistance develops in Pa following repeated antibiotic exposure. With the advent of elexacaftor/tezacaftor/ivacaftor (ETI) modulator therapy, we wished to assess Pa antibiotic resistance to beta-lactam and fluoroquinolone (FQ) antibiotics in both children receiving ETI, and those not eligible for ETI (nonETI) who continue to experience recurrent pulmonary exacerbation (PEx) and require repeated antibiotic treatment.

**Methods:**

We examined current patterns of antibiotic resistance over 3 years for a cohort of 11 children with CF having 5 or more positive sputum cultures for Pa between January 2020-December 2022 obtained from CF clinic and hospital PEx sputum samples. Chart review included: demographic data; dates of PEx/hospitalization; dates of CF clinic visits; dates and results of CF sputum cultures; and antibiotics used and dates for intravenous (IV) and oral (PO) treatment courses.

**Results:**

Six males and 5 females were reviewed (6/11 [54%] were Hispanic), with 3/11 (27%) on ETI therapy (average age 14.6 yr), and 8/11 (73%) not on ETI therapy (average age 11.6 yr). Average age at acquisition of Pa was 10.2 yrs. The average number of cultures yielding Pa over 3 years was 11 for both ETI and nonETI subjects. Average number of IV antibiotic courses was 6 for nonETI, but 0 for ETI subjects, while the average number of oral (PO) FQ courses with ciprofloxacin/levofloxacin was 6 in nonETI, and only 1 in ETI subjects. No increase in antibiotic resistance was noted against any IV antibiotic over 3 years of observation (Table), but of the 9 subjects (both ETI and nonETI) who received PO FQ, 5 (55%) were documented to have at least one nonsusceptible isolate, as shown for one 16 year old nonETI subject (Figure).

**Figure:**
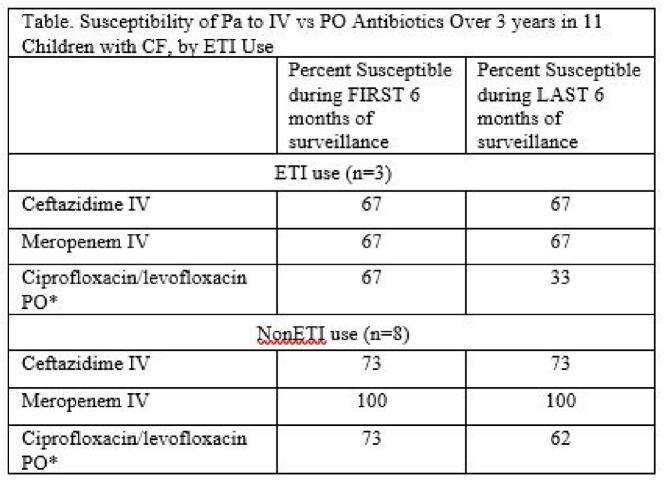

**Figure:**
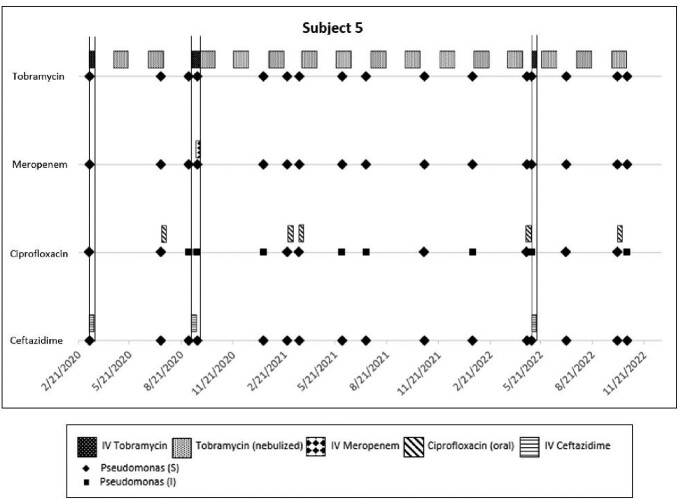

**Conclusion:**

Current antibiotic/pulmonary management of children with CF was not associated with an increased risk of IV antibiotic resistance over 3 years of observation despite repeated exposure for PEx (mean of 6 IV antibiotic courses per nonETI subject), but susceptibility to FQ decreased in 55% of those treated over 3 years following repeated FQ oral therapy courses.

**Disclosures:**

**All Authors**: No reported disclosures

